# Peach volatile emission and attractiveness of different host plant volatiles blends to *Cydia molesta* in adjacent peach and pear orchards

**DOI:** 10.1038/s41598-020-70685-9

**Published:** 2020-08-12

**Authors:** Peng-fei Lu, Hai-li Qiao

**Affiliations:** 1 The Key Laboratory for Silviculture and Conservation of the Ministry of Education, School of Forestry, Beijing Forestry University, Beijing, 100083 China; 2 Institute of Medicinal Plant Development, Chinese Academy of Medical Sciences and Peking Union Medical College, Beijing, 100193 China

**Keywords:** Entomology, Chemical ecology

## Abstract

The oriental fruit moth (OFM), *Cydia* (= *Grapholitha*) *molesta*, is a highly damaging pest; peaches are its primary host, and pears serve as post-peach secondary hosts during the late season in China. We collected volatiles from detached peach shoots and fruits, and identified them with gas chromatography–mass spectrometry (GC–MS). Antennally active compounds were identified by gas chromatography-electroantennogram detection (GC-EAD), and these were further tested in the laboratory and field. We detected consistent electroantennographic activity was for ten compounds. Significantly more *C. molesta* females were caught with a mixture of female EAD-active compounds identified from the detached matured peach fruits (nonanal, butyl acetate, 3-methylbutyl acetate, hexyl acetate, (*Z*)-3-hexenyl acetate, linalool and farnesene) than other mixtures mimicking the volatile profile from detached matured fruits or shoots. We identified a new GC-EAD active mixture from intact peach shoots composed of nonanal, (*Z*)-3-hexenyl acetate, (*E*)-β-ocimene, and 6-methyl-5-hepten-2-one. In the field test, the background odour of orchards could affect trap catches, and two peach-derived blends together with two previously known pear-derived blends were proven to be able to monitor the seasonal OFM population dispersal in adjacent orchards. These host plant blends will be effective for further designing candidate attractants for season-long *C. molesta* population dynamic monitoring.

## Introduction

Phytochemicals are important olfactory cues for moths to find hosts and lay eggs^1–4^. For some multivoltine insect species, host volatiles also play a crucial role in seasonal host shifts^5^. Substantial variations in volatiles emitted by different host plants at different phonological stages presents an enormous challenge for multivoltine insect herbivores^6–8^. To locate suitable host plants, these insects need to accurately discriminate different chemical profiles according to seasonal progression and make the correct behavioural selection^9–12^. In particular, females must lay eggs on suitable hosts to reproduce and sustain the population^13^. Therefore, comprehensive understanding of volatile emission by different host plants could provide crucial information to elucidate behavioural adaptations of these multivoltine insects to facilitate seasonal host choice.

The oriental fruit moth (OFM), *Cydia* (= *Grapholitha*) *molesta* (Busck) (Lepidoptera: Tortricidae), is native to Asia, and has become widely distributed throughout the fruit-growing areas of the world^14,15^. Its hosts include several species of stone fruit and pome fruits within the family Rosaceae. In many distributed regions such as China, peach (*Prunus persica* L.) is its primary host during the early growing season, and then they disperse into pear orchards (*Pyrus communis* L.) or apple orchards (*Malus domestica* L.) during the mid-late growing season^16,17^. Many studies indicated that populations are capable of switching freely from stone fruit orchards to pome fruit orchards throughout the growing season^17–24^. As a result, pear or apple trees planted adjacent to peach orchards seem to be more heavily damaged by OFM relative to orchards farther away from peach orchards^17^. More recently, greater attention has been devoted to developing a comprehensive understanding of *C.* m*olesta* developmental differences based on different host crops volatiles. Furthermore, host plant volatiles have been suggested as useful for development of monitoring and mating disruption tools for female moths under field conditions^25,26^.

Most studies on odorant profiles released from *C. molesta* hosts were limited to specific plant parts from a single species at one timepoint in the season. These researches mainly focused on detached young peach shoots^27,28^, fresh young peach shoots^23^ or detached peach fruits^29^. Thus far, entomologists have only started to pay particular attention to season dynamics of volatile emissions from different hosts. Preferential selection of *C. molesta* females to fresh peach and apple trees with various plant tissues during different times of the season has been reported^6^. Volatile emissions from host plants and their olfactory attraction to *C. molesta* adult were reported during a full season from peach and pear trees in situ^13^. Our research group characterized the crucial volatiles emitted from unmatured and mature pear fruit, and designed two pear-derived attractants for monitoring both sexes of oriental fruit moths under field conditions^25^; we then compared the peach and pear fruit volatiles attractive to the oriental fruit moth during the late season^26^. However, previous research results have not yet pinpointed groups of VOCs crucial to hosts attractiveness during different phenological stages, which could be used to design synthetic blends for monitoring the oriental fruit moths in the adjacent peach-pear cultivation orchards. Such knowledge would help elucidate mechanisms underlying the seasonal shift of host preference of OFMs from peach to pear, and could be used to explore the new pest management approach under the field condition.

The objectives of the study were: (1) to identify and compare the crucial VOCs active electrophysiologically to OFM from detached or intact young peach shoots and fruits, and (2) to test the behavioural responses of OFM adults to synthetic blends of these VOCs in the laboratory and in the field; finally, (3) to evaluate crucial plant volatile blends derived from peach and pear trees attractive to the oriental fruit moth in adjacent cultivation orchards of two host crops.

## Results

### Characterization of the headspace volatiles from the detached peach shoots (DS), detached matured fruits (DF) and detached unmatured fruits (DFU)

In order to understand which volatile compounds attract OFMs to peaches, we analysed the VOCs of the detached peach shoots and fruits. A total of 33 compounds were identified, which belong to the following chemical classes: hydrocarbons, alcohols, aldehydes, esters, benzene derivatives, ketones, and terpenoids (Table 1). The profiles of the detached shoots and fruits consisted of the same chemical classes; however, total emission quantities were significantly lower in shoots than in fruits across all chemical classes. In particular, five components, including octadecane, (*Z*)-3-hexen-1-ol, benzaldehyde, methyl salicylate, and (*E)*-β-ocimene, were characteristic of peach shoots (DS) and absent from either unmatured (DFU) or matured fruits (DF). Seasonal dynamics in quantitative and qualitative volatile emissions from nectarine fruits were also noted. Nonadecane, octanal, decanal, 6-methyl-5-hepten-2-one, and limonene, were characteristic of unmatured fruits (DFU) and absent from matured fruits (DF). Pentadecane, heptadecane, (*E*)-2-nonen-1-ol and 5 esters emerged in VOCs of matured peach fruits (DF). The concentrations of other VOCs, such as hexadecane, nonanal, (*Z*)-3-hexenyl acetate, and (*E, E*)-α-farnesene increased with maturation, whereas the concentrations of linalool decreased with maturation (DF). In matured fruits, esters were the dominant VOCs, and consisted of mainly 2-methylpropyl acetate, butyl acetate, 3-methyl-1-butyl acetate, hexyl acetate, methyl octanoate, and (*Z*)-3-hexenyl acetate (DF). The profiles of the two peach varieties consisted of the same chemical classes, but total emissions from nectarine variety were significantly higher than wild peach variety at all sampling dates. In particular, (*E*)-2-nonen-1-ol, 5 esters and (*E, E*)-α-farnesene were characteristic of matured nectarine variety and absent from matured wild peach variety (Table 1). (*Z*)-3-hexenyl acetate was the most abundant VOC identified in both varieties.

**Table 1:** Relative quantities of volatile compounds collected in the headspace of shoots, unmatured and matured fruits of peach.

Compounds	SO^a^	Nectarine shoot		Nectarine fruit				Wild peach fruit	
		DS^b^	IS^c^	DFU^d^ (unmatured)	DF^e^ (matured)	IFU^f^ (unmatured)	IF^g^ (matured)	DFU (unmatured)	DF (matured)
**Hydrocarbons**									
Decane*	SA							< 1	
Tridecane*	SA								< 1
Tetradecane*	SA			< 1	12	< 1	14	< 1	< 1
Pentadecane*	SA	1			24		20	100	72
Hexadecane*	SA	2	3	< 1	29	< 1	30	45	96
Heptadecane*	F	2			14		14		
Octadecane*	SA	< 1							
Nonadecane*	SA	< 1		< 1		< 1		35	63
**Alcohols**									
(Z)-3-hexen-1-ol*	SA	5							
(*E*)-2-nonen-1-ol					< 1				
2-Methyl-1-hexadecanol								< 1	< 1
**Aldehydes**									
Octanal*	F			< 1		< 1		< 1	
Nonanal*	F	< 1	14	< 1	22	< 1	26	< 1	< 1
Decanal*	F		11	< 1		< 1		< 1	
**Esters**									
2-Methylpropyl acetate*	SA				78		80		
Butyl acetate*	AO				8		8		
3-Methyl-1-butyl acetate*	SA				41		42		
Ethyl hexanoate*	TC							< 1	
Hexyl acetate*	F				23		20	< 1	
(*Z*)-3-hexenyl acetate*	SA	100	100	< 1	100	< 1	100	< 1	100
Methyl octanoate*	SA				< 1				
Butyl hexanoate	SA		8						
Hexyl butanoate	TC		10						
Hexyl 2-methylbutyrate			30						
Hexyl hexanoate			7						
**Benzenoids**									
Benzaldehyde*	SA	15							
Methyl salicylate*	SA	< 1							
**Ketones**									
6-Methyl-5-hepten-2-one *	SA		< 1	< 1		< 1		< 1	< 1
**Terpenoids**									
Limonene*	F			< 1		< 1		< 1	
(*E*)-β-ocimene*	SA	87	86						
Linalool*	F	< 1		100	42	100	45	26	< 1
(*E, E*)-α-farnesene*	SA		23	< 1	3	< 1	2		
(*E*)-β-caryophyllene*	F		< 1						

The asterisked compounds had been conclusively identified by comparison of spectra and retention times with those of an authentic standard. Compounds in bold face type elicited antennal responses in Gas chromatography-Electroantennogram detection (GC-EAD) experiments. Compounds within each class were listed according to retention times on a polar DB-Wax fused silica column. The varieties of peach species were nectarine peach of *Prunus persica* (L.) Batsch cv. Shuguang and wild peach from *Prunus persica* (L.) Batsch cv. Shenzhoubaimi. Quantities are expressed relative to the most abundant compound (set to a value of 100) in the shoots and two stages of peach fruits. The average amount ± SD of the most abundant compound collected from 100 g of plant issues (N = 5) in the different phenological stages was: Nectarine shoot) 78.79 ± 7.85 ng/h of (*Z*)-3-hexenyl acetate in detached shoots (DS); 104.13 ± 18.15 ng/hr of (*Z*)-3-hexenyl acetate in intact shoots (IS); Nectarine fruit) 6.01 ± 2.97 ng/h of linalool in unmatured detached fruits (DFU); 58.75 ± 7.91 ng/h of (*Z*)-3-hexenyl acetate in matured detached fruits (DF); 7.67 ± 3.21 ng/h of linalool in unmatured intact fruits (IFU); 63.33 ± 9.45 ng/h of (*Z*)-3-hexenyl acetate in matured intact fruits (IF); wild peach) 16.39 ± 2.26 ng/h ng/h of pentadecane in unmatured detached fruits (DFU); 29.19 ± 5.63 ng/h (*Z*)-3-hexenyl acetate in matured detached fruits (DF).

^a^*SO* source of authentic standards. The standards were obtained from Sigma-Aldrich Co., St. Louis, MO, USA (SA), Fluka Production GmbH, Buchs, Switzerland (F), Acros Organics, New Jersey, USA (AO), Tokyo Chemical Industry CO., Tokyo, Japan (TC).

^b^ DS and ^c^IS denote detached and intact peach shoots respectively.

^d^DFU and ^e^DF denote unmatured and matured detached peach fruit respectively.

^f^IFU and ^g^IF denote unmatured and matured intact peach fruit respectively.

### Antennal responses to VOCs from the detached peach shoots (DS) and detached matured fruits (DF)

**Figure 1:**
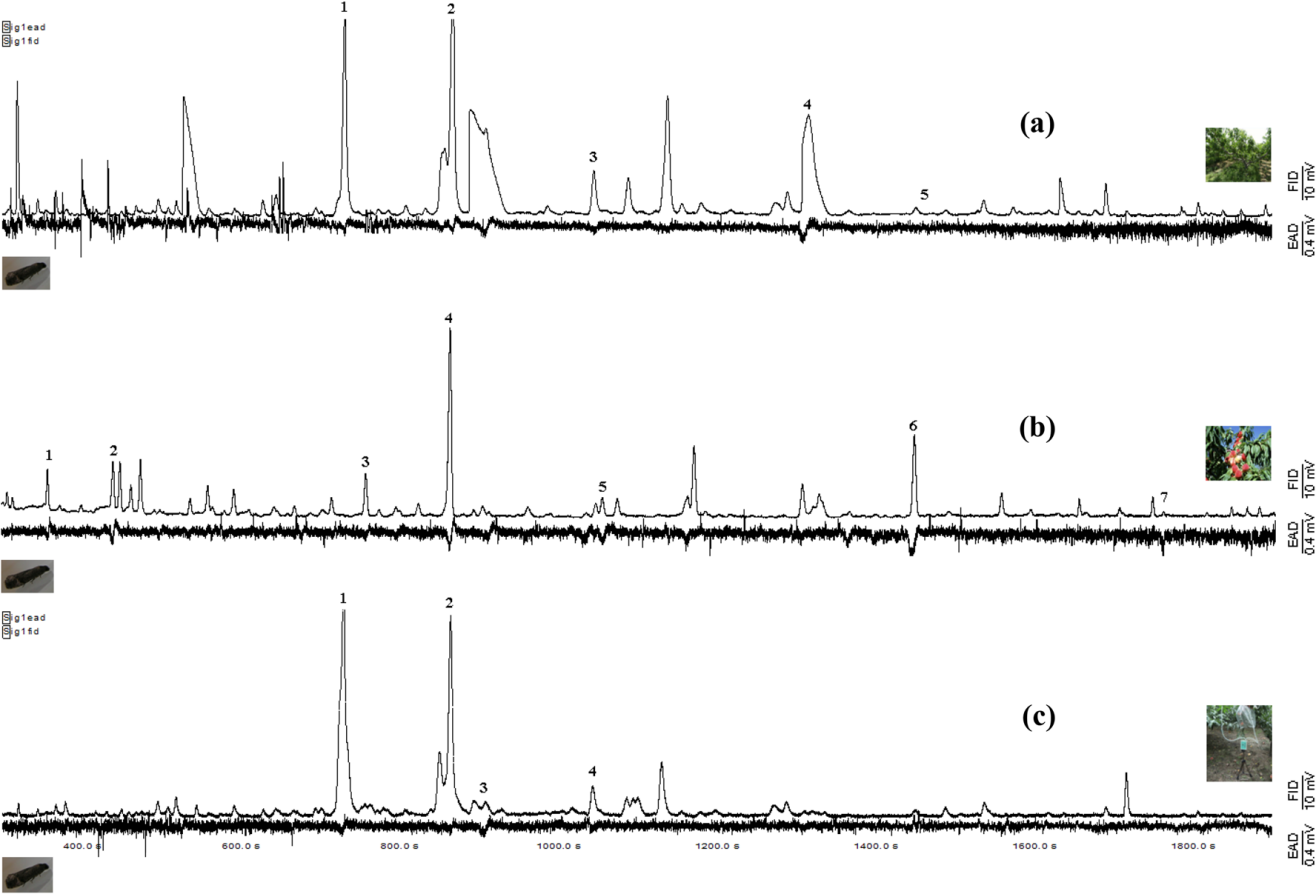
Simultaneously recorded GC-EAD responses to VOCs collected from detached shoots (DS) (trace **a**), matured detached fruits (DF) (trace **b**) and intact shoots (IS) (trace **c**) of the nectarine peach varieties Shuguang of *Prunus persica* using a polar DB-WAX capillary column. The upper trace is the flame ionization detector response (FID), and the lower displays the *Cydia molesta* female antennal response (EAD). Consistently EAD-active compounds in six different tests are shown as follows: For (**a**): (1) (*E*)-β-ocimene; (2) (*Z*)-3-hexenyl acetate; (3) (*Z*)-3-hexen-1-ol; (4) benzaldehyde; (5) linalool. For (**b**): (1) butyl acetate; (2) 3-methylbutyl acetate; (3) hexyl acetate; (4) (*Z*)-3-hexenyl acetate; (5) nonanal; (6) linalool; (7) farnesene. For **(c)**: (1) (*E*)-β-ocimene; (2) (*Z*)-3-hexenyl acetate; (3) 6-methyl-5-hepten-2-one; (4) nonanal.

In total, ten compounds from the headspace of detached shoots and fruits elicited antennal responses by OFM females: (*E*)-β-ocimene, (*Z*)-3-hexenyl acetate, (*Z*)-3-hexen-1-ol, benzaldehyde, linalool, butyl acetate, 3-methylbutyl acetate, hexyl acetate, nonanal, and (*E, E*)-α-farnesene (Fig. 1a,b). The EAD-active VOCs differed in quality and quantity between VOCs of detached shoots and fruits. However, (*Z*)-3-hexenyl acetate and linalool were common components from detached peach shoots and fruits (Fig. 1a,b). Esters were frequently present among GC-EAD-active volatiles in matured peach fruits. Three components specific to detached shoots elicited antennal responses from OFM females, which were (*Z*)-3-hexen-1-ol, benzaldehyde, and (*E*)-β-ocimene. Five components specific to matured fruits elicited antennal responses from OFM females, which were butyl acetate, 3-methylbutyl acetate, hexyl acetate, nonanal and farnesene, (Fig. 1a,b).

### Comparative analysis of the headspace volatiles from the detached (DS) and intact peach shoots (IS)

The profiles of the detached and intact peach shoots consisted of the same chemical classes; however, quantitative and qualitative difference were noted. In particular, eight components were characteristic of detached peach shoots, which comprised several hydrocarbons, (*Z*)-3-hexen-1-ol, benzaldehyde, methyl salicylate, and linalool; these are likely to be wound-associated volatiles produced after cutting the shoots from plants. Other components were characteristic of intact peach shoots, which included decanal, several esters, (*E, E*)-α-farnesene, (*E*)-β-caryophyllene, 6-methyl-5-hepten-2-one (Table 1). The detached and intact shoots share two common GC-EAD-active VOCs, (*E*)-β-ocimene and (*Z*)-3-hexenyl acetate. (*Z*)-3-hexenyl acetate was the most abundant GC-EAD active VOC identified in both tissues (Fig. 1a,c). A newly identified component in intact shoots, 6-methyl-5-hepten-2-one, can elicit moth EAG response, although it is present at minor quantities (Fig. 1c). No differences were found between the profiles of the detached (DF) and intact peach matured fruits (IF) (Table 1).

### Wind tunnel bioassays

**Figure 2:**
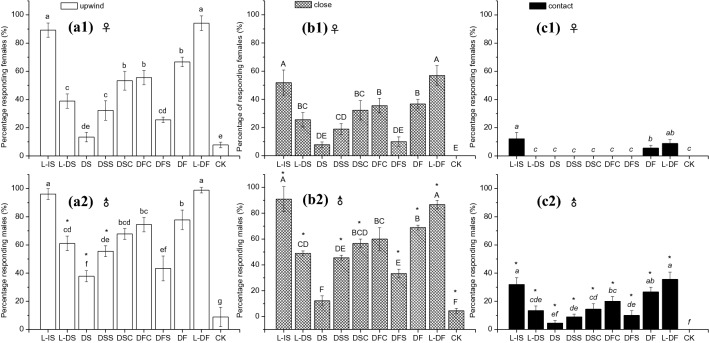
Attraction of mated *Cydia molesta* females (**a**) and males (**b**) in a wind tunnel to synthetic VOC mixtures mimicking the volatile profile from detached shoots (DS), matured detached fruits (DF) of the peach varieties Shuguang of *Prunus persica*, their subsets (DSS, DSC, DFS, DFC) and a hexane control (CK). Individual moths were scored for the following behaviours: (1) departure from the release cage and flight upwind (**a1** and **a2**, for females and males); (2) arrival within 10 cm of the VOC source (**b1** and **b2**, for females and males), and (3) landing on the source. (**c1** and **c2**, for females and males). The ratios are shown in Table 2. Within columns with same colour, means ± SD followed by different letters are significantly different (one-way ANOVA followed by Tukey's multiple comparison test, *P* < 0.05). Significant differences in three flight behaviours between both sexes were analysed by the Mann–Whitney *U*-test (*P* < 0.05; asterisked column, significant difference between both sexes).

The peach variety was nectarine peach of *Prunus persica* (L.) Batsch cv. Shuguang. The six blends mimicking the volatile profile were from detached shoots (DS), detached matured fruits (DF) and their subsets (DSS, DSC, DFC, DFS). “L” means living and freshly detached peach tissue. Similar to the results in field experiment 1, the attractiveness of complete synthetic mixture (DS) was significantly lower than the two partial mixtures (DSC, DSS). The attractiveness of freshly detached peach shoots from 1-year-old potted peach plants (L-DS) and DSC, DSS was not significantly different. The fresh intact peach shoots from potted peach plants (L-IS) were shown to be one of the most attractive to both sexes in the wind tunnel (Fig. 2). Of the females, 89% flew upwind (Fig. 2a1), 52% arrived within 10 cm of the source (Fig. 2b1) and 12% landed on the source (Fig. 2c1); of the males, 96% showed upwind orientation (Fig. 2a2), 91% arrived within 10 cm of the source (Fig. 2b2) and 32% landed on the source (Fig. 2c1).

The attractiveness of complete synthetic mixture (DF) was significantly higher than the partial mixtures (DFS) and similar to another partial mixture (DFC). The freshly detached peach fruits (L-DF) were shown to be among the most attractive to both sexes in the wind tunnel (Fig. 2); of the females, 94% flew upwind (Fig. 2a1), 57% arrived within 10 cm of the source (Fig. 2b1) and 9% landed on the source (Fig. 2c1). In contrast, 99% of males showed upwind orientation (Fig. 2a2), 87% arrived within 10 cm of the source (Fig. 2b2), and 36% landed on the source (Fig. 2c2).

Males were more strongly attracted than females to fresh plant tissues and synthetic mixtures. L-IS, L-DF and DF enticed both sexes to land on the lures (Fig. 2c1,c2). The other samples only stimulated males to reach and contact the source (Fig. 2c2).

### Field experiment 1

**Figure 3:**
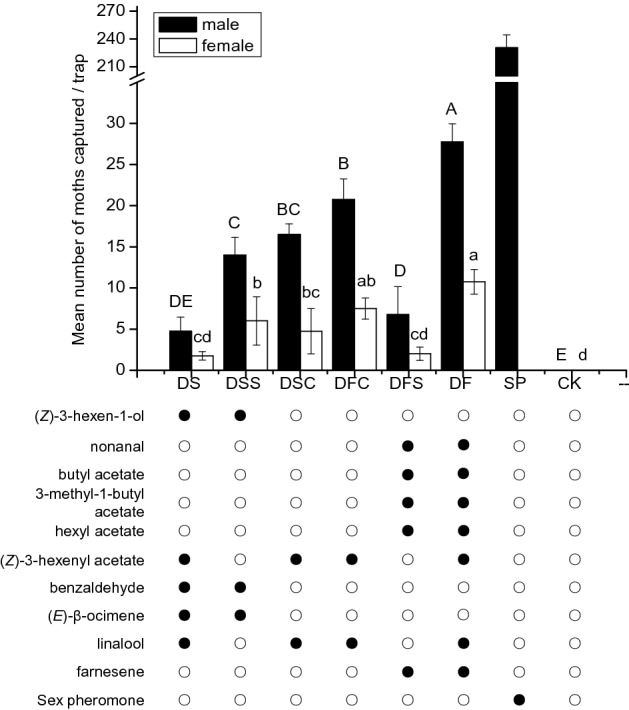
Mean total number ± SD of *Cydia molesta* males and females captured in each trap (N = 6) in a pear orchard on 5–15 September, 2018. Each lure was baited with rubber septa with the synthetic VOC mixtures based on headspace VOC composition from detached shoots (DS), detached matured fruits (DF) of the peach varieties Shuguang of *Prunus persica*, their subsets (DSS, DSC, DFS, DFC), sex pheromone (SP) and a hexane control (CK). The ratios are shown in Table 2. The experiment was conducted at an experimental orchard at the Institute of Forestry and Pomology (IFP), Beijing Academy of Agricultural and Forestry (BAAF), Beijing, China. Different letters (capital letters for males captured and small letters for females) on bars indicate significant differences (one-way ANOVA followed by Tukey's multiple comparison test, *P* < 0.05).

Significantly more OFM males were caught in traps baited with the blends mimicking the volatile profile from detached matured fruits (DF) (nonanal, butyl acetate, 3-methylbutyl, acetate hexyl acetate, (*Z*)-3-hexenyl acetate, linalool and farnesene in a 22:8:41:23:100:42:3 ratio) than the control or traps baited with the other five blends; this was followed by the partial mixtures from peach fruit (DFC) (Fig. 3). Surprisingly, the lowest attraction to males among all six blends was with a complete blend mixture from detached peach shoots (DS) ((*Z*)-3-hexen-1-ol, (*Z*)-3-hexenyl acetate, benzaldehyde, (*E*)-β-ocimene, linalool in a 5:100:15:87:1 ratio). However, the two partial mixtures, DSC and DSS, derived from detached peach shoots were significantly more attractive to the males than the complete mixture DS (Fig. 3). Fewer females were trapped in all mixtures relative to males. DF caught more OFM females than the control or the traps baited with the other five blends, followed by DFC with no significant difference between traps. Similar to males, the lowest attraction to females among all six blends occurred with DS (Fig. 3).

### Field experiment 2

In order to screen effective synthetic blends which could be used for season-long monitoring of OFM in the different phenological stages**,** four plant-derived mixtures were further evaluated in adjacent orchards of peach and pear; IS and DF blends were based on peach, and JM and HJ blends were based on pear^25^. Each field test was conducted according to phenological stages of the two host plants.

**Figure 4:**
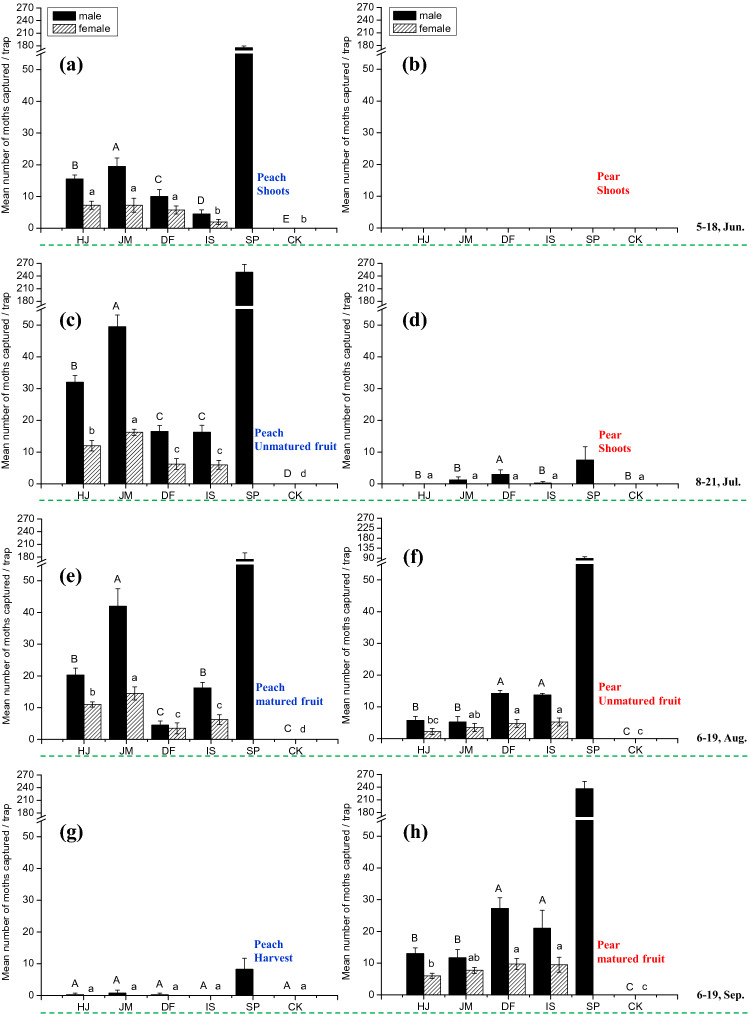
Mean total number ± SD of *Cydia molesta* males and females captured in each trap (N = 6) in peach and pear orchards from June to September 2019. (**a**) and (**b**), the peach orchard and the pear orchard during 5 June–18 June, respectively; (**c**) and (**d**), the peach orchard and the pear orchard during 8 July–21 July, respectively; (**e**) and (**f**), the peach orchard and the pear orchard during 6 August-19 August, respectively; (**g**) and (**h**), the peach orchard and the pear orchard during 5 September–19 September, respectively. Each trap was baited with a rubber septum with synthetic VOC mixtures corresponding to those emitted by pear and peach fruit, peach shoots, a hexane control (CK), and sex pheromone (SP). The ratios are shown in Table 3. The two varieties of pear species were Jimi (JM) of *Pyrus bretschneideri*, Huangjin (HJ) from *Pyrus pyrifolia*. The peach variety was Shuguang of *Prunus persica*. The experiment was conducted at an experimental orchard at the Institute of Forestry and Pomology (IFP), Beijing Academy of Agricultural and Forestry (BAAF), Beijing, China. Different letters (capital letters for males captured and small letters for females) on bars indicate significant differences (one-way ANOVA followed by Tukey's multiple comparison test, *P* < 0.05).

During the growing stage for early shoots of the two plant species between 5 and 18 June, the OFM population was apparently present in the peach orchard based on the number of male moths trapped with the sex pheromone (Fig. 4a); in contrast, they could not be found in the pear orchard (Fig. 4b). Significantly more OFM males were caught in traps baited with the blends mimicking the volatile profile from pear variety JM (1-hexanol, nonanal, ethyl butanoate, butyl acetate, ethyl hexanoate, hexyl acetate, hexyl butanoate, and farnesene in a 1:1:100:70:7:5:1:4 ratio) and variety HJ (nonanal, ethyl butanoate, 3-methylbutyl acetate, ethyl hexanoate, hexyl acetate, farnesene with a 1:100:1:32:1:2 ratio) than the control and the other peach derived lures (Fig. 4a). Compared with peach shoot derived lures (IS), peach fruits (DF) were significantly more attractive to males (Fig. 4a). Only a few females were trapped by four mixtures with no significant differences between traps aside from blend IS (Fig. 4a).

A larger OFM population was present in the peach orchard during the early fruiting stage for peach and shooting stage for pear between 8 and 21 July than was present during the first stage, as indicated by the number of male moths trapped with the sex pheromone (Fig. 4a,c). A significantly larger OFM population was present in the peach orchard than in the pear orchard (Fig. 4c,d). As in first stage, traps in the peach orchards baited with blends of the JM and HJ varieties caught significantly more OFM males and females than the other traps (Fig. 4c). In the pear orchard, few OFMs males could be caught with sex pheromones; no females were caught in the traps (Fig. 4d).

During the late fruiting stage for peach and early fruiting stage for pear between 6 August and 19 August, greater numbers of OFMs males were found in peach orchards, as monitored by sex pheromone (Fig. 4e,f). During this time period in the peach orchard, traps baited with blends of the JM varieties caught significantly more OFM males and females than the other traps (Fig. 4e). Compared with peach fruits (DF), peach shoot derived lures (IS) were significantly more attractive to males (Fig. 4e). In the pear orchard, the attractiveness of two peach-derived lures (DF and IS) to male was significantly higher than two pear-derived lures (HJ and JM). Only a few females were trapped by the four mixtures, and with no significant difference between traps aside from blend HJ (Fig. 4f).

During the peach harvest and late fruiting stage for pear between 5 and 19 September, a larger OFM population was present in the pear orchard relative to the peach orchard, based on the number of male moths trapped with sex pheromone (Fig. 4g,h). In the pear orchard, the attractiveness of two peach-derived lures (DF and IS) was significantly higher to males than were the two pear-derived lures (HJ and JM). Compared with female catches during the third stage, more females were trapped by 4 mixtures with no significant differences between traps aside from blend HJ (Fig. 4h).

## Discussion

Herbivores with multiple generations per year are confronted with many different volatiles at different concentrations and in different combinations. Thus, insect herbivores need to discern relevant host odour cues during the different phenological stages and against high background noise^30,31^. *C. molesta,* a typical multi-voltine species, utilizes peach and pear as its respective primary and secondary hosts during different periods of the growing season. Our studies indicated that headspace volatiles from peach-shoot, peach fruits, and pear fruits^25^ differed greatly in quality and quantity. OFM should possess an odour coding system which is sufficiently flexible to accommodate variation in volatile profiles. OFM is not equally attracted to these different plant-derived blends. Similar results had been found in several herbivore species^2,7^. Multivoltine insect species with multiple generations per year exploit several plant species as their hosts in different phenological stages. It is strategically important for insect species to optimize their resources in order to complete the life cycle. These data are crucial to develop semiochemical-based synthetic lures for use in the field^10,32^.

Although differences have been found between the profiles of headspace volatiles from peach intact shoots and fruits, the blends from the various phenological stages still share a common set of chemicals, including (*Z*)-3-hexenyl acetate, nonanal, decanal, 6-methyl-5-hepten-2-one, (*E, E*)-α-farnesene; most of these components did elicited EAG response (Table 1). A recent study suggested the switch of OFMs from the primary host peach to a secondary host such as pear or apple could be facilitated by the presence of a common set of five aldehydes, and suggested which would play an essential role in female attraction to host plants^13^. Similar results had been found in other herbivore species^33,34^. This is also an important strategy for multi-voltine herbivores to modulate their behaviour according to chemical and taxonomic similarities across host plants.

Quantitative as well as qualitative differences can have a significant effect on OFM behavioural responses. Firstly, the whole plant volatile blends are more attractive than the sum of their components. In the study, the complete mixtures of peach fruits (DF) were significantly more effective than the two subset mixtures (DFS, DFC) in the field and in the wind tunnel assay (Figs. 2, 3). Similarly, the complete JM mixture proved to be the most effective in the field test using the lure derived from matured pear fruits^25^, followed by the complete HJ mixture; the complete mixtures were significantly more effective than the partial mixtures (JMS JMC, HJS, HJC)^25^. A previous study has suggested that appropriate blends or combinations of volatiles could elicit stronger behavioural responses than with single compounds^3^. One of the earliest experiments showed that none of the individual components ((*E*)-3-hexen-1-ol, (*Z*)-2-hexen-1-ol, (*E*)-2- hexen-1-ol or (*E*)-2-hexenal) from potato were attractive to Colorado beetle, *Leptinotarsa decemlineata* (Coleoptera: Chrysomelidae), but attraction occurred once these volatiles were combined as a mixture^35^. The results suggest that individual components or mixtures made of a too narrow subset of host volatiles are always perceived as non-host cues, but volatiles combined together are perceived as a suitable host attractant. On the other hand, the proportions of individual components are also crucial for host location. Specific odours from certain host plants can be used by some insects as recognizable olfactory signal^36–38^; however, for many herbivore insects, active compounds eliciting antennal response are almost relatively ubiquitous. EAD-active VOCs in our study, such as (*E*)-β-ocimene, (*Z*)-3-hexenyl acetate, (*Z*)-3-hexen-1-ol, linalool, nonanal and (*E, E*)-α-farnesene, could also be identified from many plant species. Suitable host plants emitting only common VOCs can likely be recognized through subtle mixture proportions, thereby providing detailed information for the herbivores to locate their hosts; this has also been indicated in many earlier studies as well^39–41^.

By comparing the attractiveness of fresh plant tissues from intact and detached peach shoots in the wind tunnel, we found the intact shoots were significantly more effective than detached shoots. Two subset mixtures (DSS, DSC) from detached peach shoots were also more attractive than complete mixtures of detached peach shoots (DS). We hypothesized that collection of volatiles from cut young peach shoots may distort the chemical information from intact peach shoot volatiles and affect greatly the olfactory orientation of OFM in the field. Four components, consisting of (*Z*)-3-hexen-1-ol, benzaldehyde, methyl salicylate, and linalool, were characteristic of detached peach shoots. An early study found 22 compounds in the headspace of excised peach shoots^27^. (*Z*)-3-hexen-1-ol, benzaldehyde and methyl salicylate have also been detected in their study. The four components characterized in detached shoots in our study have not been found in the headspace of intact shoots^23^. Similar to our results, farnesene was detected in the study, although as a different isomer^23^. Methyl salicylate can elicit larger EAG responses in many tortricids, such as *Lobesia botrana*^42^, *C. strobilella*^43^, *C. pomonella*^44^. Methyl salicylate is shown to be a common plant compound involved in stress signalling^45^; it attracts natural predators^46^ and at same time discourages female oviposition^47^. (Z)-3-Hexen-1-ol, methyl salicylate and linalool were detected from Herbivore-damaged *Aquilaria sinensis* plants, which discourages oviposition of the herbivore *Heortia vitessoideson*; on the contrary, these induced components likely act as important cues for the predator *Cantheconidea concinna* to locate its prey herbivore^48^. Thus, we deduced that these components characteristic of detached shoots should be wound-associated volatiles emerging after artificial cutting off shoots from twigs. These emerging components were associated with plant stress caused by manual damage, and it could indicate a lower quality of either host or non-host. Contrarily, decanal, (*E, E*)-α- farnesene, 6-methyl-5-hepten-2-one, (*E*)-β-caryophyllene, and several esters were characteristic of intact peach shoots, and these should be representative healthy and suitable host plants. The fact that intact peach shoots were more attractive than detached shoots suggested that active avoidance of odours indicative of lower host quality or ratio distortion due to experimental conditions and even nutritionally unsuitable hosts appears to be an important part of the insect host location process. Although as a redundant chemical signal, (*Z*)-3-hexen-1-ol and benzaldehyde are still behavioural related components^27,28^, which indicated the plasticity of the OFMs host plant recognition system. In this study, males had stronger behavioural responses than females toward host plant synthetic lures both in the laboratory and the field; this was also observed in some earlier studies^49,50^, which together indicate that each sex employs different strategies.

An important task for males is to find females to mate. Males use plant VOCs to distinguish environments where they are more likely to find females^23^. Males flying in search of females are adapted to locate a point-source of sex pheromone. By contrast, females prefer to look for a suitable oviposition site, and thereby need to consider more factors; in addition to plant VOCs, females will consider resource fitness and plant background under field conditions. Thus, females searching for suitable oviposition sites may not be as strongly attracted to point sources of plant VOCs. All these hypotheses have been validated in our wind-tunnel study; although females were attracted by synthetic lures, the performance of males exceeded that of females, especially at close range. Similar results for OFM were found in our early study^51^. In addition, this observation was noted for the codling moth, *C. pomonella,* which is an important pest of apple^52^. Female codling moths frequently flew upwind toward branches with green apples over several meters, but seldom contacted the apples.

Field evaluation of four host plant VOC blends indicated that the background odour of the orchard interferes with the attractiveness of synthetic blends. From the first to the third stage, the two pear-derived lures caught significantly more OFM males than other lures in peach orchards, while the two peach-derived lures were more attractive than other lures from the third to fourth stage in the pear orchard. Interestingly, in the peach orchard, the fruit odour mimic (DF) was more attractive than shoot derived lures (IS) during the early season when natural fruit were not yet developed. Accordingly, the attractiveness of peach shoot derived lure (IS) surpassed peach fruit derived lures (DF) during the third stage when no new shoots were available. In the pear orchard, peach derived lures (DF, IS) are stronger attractants than pear derived lures (HJ, JM) during the third to fourth stage. All the results indicated the background odour of orchards could affect trap catches; thus, the blends derived from one plant species should be offered in the context of other plant species. These data provide important insights for furthering the design of candidate attractants for *C. molesta* during different seasons and among different plant types.

## Methods

### Insects

The OFMs larvae were collected in late June from infested peach shoots, *Prunus persica* L. Batsch cv. Dajiubao from an orchard at the Institute of Forestry and Pomology (IFP), Beijing Academy of Agriculture and Forestry (BAAF), Beijing, China (39° 58′ N, 116° 13′ E). Young larvae were mass-reared on apple, *Malus domestica* L. Borkh. cv. Hongfushi in a container (27 cm diameter, 13 cm height), and then reared individually in smaller glass containers (2.5 cm diam., 8 cm height) from 4th instar to eclosion. Upon emergence, adults were maintained in a bell-shaped glass container (diam. of the two openings 6 and 15 cm, 41 cm height), and were provided with water-soaked cotton (15% honey solution) from the small side^23^. Moths were reared in the laboratory for three generations before testing^23^. Two- to three-day-old mated females were used in electrophysiological experiments (GC-EAD) and wind-tunnel bioassays. In order to ensure mating, groups of 20 newly emerged females and 30 males were kept in the same cage for two scotophases. The adult moths were used only once and were not exposed to synthetic odour sources before the bioassay. Conditions of the rearing chamber were 24 ± 1 °C and 65–70% RH under a photo:scoto regime of 16 L:8 D, with the photophase starting at 05:00 a.m.

### Chemicals

The identities of compounds from headspace volatiles were verified by comparison with synthetic standards. Compounds were purchased from Sigma-Aldrich Co. (St. Louis, MO, USA), Fluka Production GmbH (Buchs, Switzerland) and Tokyo Chemical Industry Co. (Tokyo, Japan).We used this mixture of farnesene isomers from Sigma-Aldrich Co. for our EAG, wind tunnel and field studies (referred to “farnesene”), which included (*E,E*)-alpha-farnesene (49%) also (*E*)-beta-farnesene (26%), (*Z*)-beta-farnesene (18%), and (Z*,E*)-alpha-farnesene (7%). (*E*)-β-ocimene (60%) was a gift from Prof. van Loon. J. J. A. (Wageningen University, Netherlands). Compounds that did not elicit antennal responses, and for which no standards were available, were tentatively identified using the NIST-database.

### Plant materials

Ten-year-old peach trees were cultivated in a 7-hectare peach orchard at the IFP. Two varieties commonly grown in the Beijing area were selected: wild peach of *Prunus persica* (L.) Batsch cv. Shenzhoubaimi and nectarine peach of *Prunus persica* (L.) Batsch cv. Shuguang. The nectarine variety is significantly more susceptible to OFM than the wild peach variety in the field. During the tests, no insecticides or any specific treatment against OFM were used in the orchard. A pear orchard of 6.5-hectare was located in the IFP at a distance of ~ 30–50 m to the peach orchard.

### Collection of VOCs from detached and intact plant tissues

The peach shoots (~ 30 cm long) were cut from the designated trees, sealed with liquid paraffin (Fluka Production), and transferred to the nearby laboratory^53^. Unmatured and matured fruits were harvested according to the phenological development of corresponding varieties in the Beijing area^54^, which were healthy and were picked within 20 min that preceded the sampling of VOCs or wind-tunnel assays. A push–pull system was used to collect headspace VOCs. Air originating from a vacuum pump (Qianxi Air Company, Beijing, China) was filtered through an activated-charcoal filter, then pushed into a 2,000 mL glass jar loaded with peach shoots (~ 220 g) and fruits (~ 1,500 g) for extraction; air was then passed through a sorbent cartridge (Porapak Q, 50 mg, 80/100 mesh, Supelco, Bellefonte, PA, USA), and finally was pulled with Teflon tubing to a small pump (Beijing Institute of Labor Instruments, China) described in our earlier study^23^. Both the push and pull flow rate were at 300 mL/min. The sorbents were placed into a glass tube (10 cm long, 0.5 cm inner diam.) with glass wool plugs on both sides, which were prepared before collection. The samples were collected for 8 h at 24 ± 1 °C and 65–70% RH. Hexane (500 μL, HPLC grade, Sigma-Aldrich) was used to elute the sorbent cartridge at room temperature. Odour samples were collected from clean glass jars without any plant material as a control. Samples were collected five times per treatment.

For quantitative analyses, internal standard (0.5 μg ethyl pentanoate, 98%, Sigma-Aldrich Co., St. Louis, MO, USA) was added into the solvent prior to elution of the sorbent. This compound was not detectable in the headspace of the peach tissue studied here in our preliminary analyses. After elution, each sample volume was reduced to 50 μL by using a slow stream of nitrogen and then analysed. If not used immediately, extracts were sealed in glass vials and stored at − 18 °C until needed.

In addition, headspace volatiles from intact young peach shoots and fruits were collected in the field directly, which permitted us to avoid collection of any volatiles induced from artificial damage caused by cutting of plant tissues, and to determine qualitative and quantitative differences in volatiles collected in the laboratory vs. in the field. This method was similar to that described by Sun et al.^55^, but with minor modifications. Firstly, the whole peach shoots (~ 30 cm long) were enclosed in a plastic oven bag (40.6 × 44.4 cm; Reynolds roasting bag, Richmond, Virginia, USA) and then sealed with self-sealing strips around the stem. The compressed air was pushed into a water bubbler (500 mL) for humidification and then a freshly activated charcoal filter for purification. The moisturized and filtered air was pushed into the bag at a rate of 300 mL/min. Volatiles were trapped in a sorbent cartridge (Porapak Q, 50 mg, 80/100 mesh, Supelco, Bellefonte, PA, USA). The sorbents were held between plugs of glass wool in a glass tube (10 cm long, 0.5 cm inner diam.). The glass tube was connected with Teflon tubing to pull the airstream by a small pump at a rate of 300 mL/min (Beijing Institute of Labor Instruments, China). Each collection lasted for 8 h and was replicated five times. Volatiles were eluted as described above. The odour samples were collected from clean roasting bags as a control.

### Gas chromatography–mass spectrometry (GC–MS)

Chemical and electrophysiological analyses was similar to that described by Lu et al.^23^. Headspace VOCs of peach shoots, unmatured or matured fruits and mixtures of synthetic compounds were identified on an Agilent Technologies 5973 MS (Agilent) with electron impact (EI) ionization (70 eV), which was interfaced with an Agilent Technologies 6,890 N GC (Agilent) equipped with polar DB-WAX fused-silica column (30 m × 0.25 mm ID, 0.25 µm film, J&W Scientific Inc., Folsom, CA, USA) or nonpolar DB-5 fused-silica column (30 m × 0.25 mm ID, 0.25 µm film, J&W Scientific Inc., Folsom, CA, USA). Oven temperature program: 1 min at 50 °C, then 3 °C/min to 120 °C, then 10–240 °C and finally held 10 min at 240 °C. Helium at 1.0 mL/min was used as the carrier gas. The temperatures of the ion source and of the interface were 230 °C and 280 °C, respectively. The emission current was 34.6 µA. Injections were made in the splitless mode. Identification of VOCs were verified by comparison with authentic standards.

### Gas chromatography-electroantennogram detection (GC-EAD)

An Agilent Technologies 6,890 N GC with a flame ionization detector was interfaced with an electroantennogram apparatus (Syntech, Kirchzarten, Germany). Both column type and oven temperature program were the same as in the GC–MS analysis. The outlet of the GC column was split in a 2:1 ratio between a cut antenna of an OFM female and the flame ionization detector (FID). Nitrogen was used as the carrier gas (1.0 mL/min). The antennae were excised using micro-scissors from heads of insects and then a few segments from the tips of antennae were clipped off. The cut antenna was mounted on two metal electrodes using conductive gel (Spectra 360, Parker Lab, NJ, USA), and then the electrode holder was inserted into the EAD probe^23^. Compounds eluting from the capillary column were delivered to the mounted antenna through charcoal-filtered and humidified air stream. The antennal and FID signals were amplified and recorded simultaneously using Syntech software (GC-EAD 32, version 4.4). Each sample was tested 6 times on 6 different antennae. Each tested antenna was derived from a different female. Six successful recordings were necessary for each treatment. Compounds eliciting repeatable antennal responses in all the six runs were regarded as active. Identities of EAD-active compounds were verified by comparison of mass spectra and retention times with those of synthetic standards.

### Wind tunnel assay

In field experiment 1, the two DSC and DSS were derived from detached peach shoots, and were more attractive to moths than the complete DS mixture; this led to a hypothesis that volatiles collection from cut young peach shoots may distort the chemical information from intact peach shoot volatiles and greatly affect the olfactory orientation of OFM in the field. Thus, we added three odour resources in the wind tunnel assay as follows: (1) L-IS: fresh intact shoots tips of peach from 1-year-old potted peach plants (~ 220 g, 40 cm long), (2) L-DS: fresh detached shoots of 10-year-old peach (~ 220 g, 40 cm long), and (3) L-DF: freshly detached fruits of peach (~ 220 g, 8–10 cm diam.). All the fresh plant tissues were freshly cut and immediately transferred into the laboratory for bioassays. Synthetic blends were loaded in rubber septa, with the predominant VOC in the mixture was dosed at 0.5 mg. The septum loaded with one of the mixtures was placed on a holder in the centre of the tunnel 10 cm from the upwind end.

The parameters of the laboratory wind tunnel were described in our earlier study^23^. Moths were sexed and then were transferred into the test room at 23 ± 2 °C and 50–70% RH 2 h before experiments. Tests began 2 h before the beginning of the scotophase and lasted 3 h. A small metal screen release cage (7 cm diam., 9 cm height) with a side door containing batches of ten mated moths was placed at the downwind end of the tunnel, 30 cm above its floor and ~ 140 cm from the VOC source. Once tests began, the side door facing the upwind end of the tunnel was opened to allow the moths to fly against the wind towards lures. Each batch of ten moths was tested for 20 min, and six batches of moths were used per day. Each treatment was tested with nine batches of moths on different days^23^. Moths were used only once. Individual moths were scored for the following behaviours: (1) departure from the release cage and flight upwind; (2) arrival within 10 cm of the VOC source, and (3) landing on the source.

### Field experiment 1

Attractiveness of volatile mixtures mimicking the volatile profile from peach were tested in the pear orchard. The pear orchard was chosen with the aim of avoiding full overlap between the background odour and synthetic blends. VOCs from the detached peach shoots (DS) and the detached matured peach fruits (DF) of the nectarine variety which elicited antennal responses based on GC-EAD analyses were formulated in blends for the field tests. In order to screen for volatiles that were essential for moth attraction, the following synthetic blends were created that included compounds common to DS and DF or specific to either source: (1) DS mimic; (2) DF mimic; (3) DSC: components common to both sources but mixed in DS ratios; (4) DFC: components common to both sources but mixed in DF ratios; (5) DSS: VOCs specific to DS; and (6) DFS: VOCs specific to DF. A glossary of abbreviations for the different plant treatments in the manuscript are shown in the online Supplementary Table S1. Six blends of synthetic compounds were prepared in the ratios of GC-EAD-active VOCs as emitted by the corresponding plant tissue and subsets (Table 2).

**Table 2:** Components and blend ratios for each blend used in the field experiment 1.

Compounds^a^	Amount loaded on rubber septum in six varieties^b^ (mg)					
	DS	DSS	DSC	DFC	DFS	DF
**Alcohols**						
(*Z*)-3-hexen-1-ol	5	5				
**Aldehyde**						
Nonanal					22	22
**Esters**						
Butyl acetate					8	8
3-Methylbutyl acetate					41	41
Hexyl acetate					23	23
(*Z*)-3-hexenyl acetate	100		100	100		100
**Benzenoids**						
Benzaldehyde	15	15				
**Terpenoid**						
(*E*)-β-ocimene	87	87				
Linalool	1		1	42		42
Farnesene^c^					3	3

^a^In order of elution during gas chromatography within each class.

^b^The peach variety was nectarine peach of *Prunus persica* (L.) Batsch cv. Shuguang. The six blends were based on composition of headspace volatile organic compounds (VOCs) from detached shoots (DS), mature detached fruits (DF) and their subsets (DSS, DSC, DFC, DFS).

^*c*^Farnesene = mixture of (*E,E*)-alpha-farnesene (49%), (*E*)-beta-farnesene (26%), (*Z*)-beta-farnesene (18%), and (Z*,E*)-alpha-farnesene (7%).

The trials were carried out in a 6.5-hectare pear orchard at the IFP with a history of OFM infestation. The formulations of the lures were same proportion as in the corresponding natural blends, with 100 mg of the most abundant compound. The mixtures of compounds were prepared 1–2 h prior to bioassays in the field trial and then added into sleeve-type rubber septa (10 mm depth, 6 mm inter diam), and finally fixed upward on the bottom of sticky delta trap (35 cm long × 20 cm high × 20 cm wide, Geruibiyuan Technology Company, Beijing, China) approximately 1.5 m above the ground in the dusk^23^. The OFM-flight intensity peaks occurred during the time. Unbaited traps (HPLC-grade hexane, Sigma-Aldrich) were used as controls. OFM sex-pheromone lures (Geruibiyuan Technology Company, Beijing, China) were used to evaluate the population density in the field. The trials were carried out in a randomized complete-block design. Six blocks were set up in the orchard at least 120 m apart. In each block, each treatment was repeated once and was set up at a distance of at least 30 m to minimize interference between traps. Captured OFMs were recorded once per day. The data of captures over a cumulative 14-day-period were subjected to data analysis. The field test was conducted from early to middle September 2018 in pear orchards, during which the adults of OFM are most active in the Beijing area.

### Field experiment 2

In order to identify effective synthetic blends to be used for season-long monitoring of the oriental fruit moth in the different phenological stages of host plants, four plant-derived mixtures derived from two host plants were further evaluated in adjacent orchards of two host crops. The treatments consist of four blends: (1) IS: intact peach shoots mimic blend, (2) DF: detached matured peach fruit mimic blend, (3) JM: detached matured pear fruit mimic blend derived from JM variety, and (4) HJ: detached matured pear fruit mimic blend derived from HJ variety. The two pear varieties were Huangjin (HJ) from *Pyrus pyrifolia* and Jimi (JM) of *Pyrus bretschneideri*. Formulation of the two pear blends was based on an earlier study^23^. Pear blends were prepared according to the same criteria as in this study. Ratios of the four blends ratios of synthetic compounds are shown in Table 3. The field test was conducted from June to September 2019 in a 7-hectare peach orchard and a 6.5-hectare pear orchard at the IFP with ~ 30–50 m open space between a planting with a history of OFM infestation. All other details of the experiment were the same as for field experiment 1 (Table 3).

**Table 3:** Components and blend ratios for each blend used in the field experiment 2.

Compounds^a^	Amount loaded on rubber septum in six blends^b^ (mg)			
	HJ	JM	DF	IS
**Alcohols**				
1-Hexanol		1		
**Aldehydes**				
Nonanal	1	1	22	14
**Esters**				
Ethyl butanoate	100	100		
Butyl acetate		70	8	
3-Methylbutyl acetate	1		41	
Ethyl hexanoate	32	7		
Hexyl acetate	1	5	23	
(*Z*)-3-hexenyl acetate			100	100
Hexyl butanoate		1		
**Ketones**				
6-Methyl-5-hepten-2-one				1
**Terpenoids**				
(*E*)-β-ocimene				86
Linalool			42	
Farnesene^c^	2	4	3	

^a^In order of elution during gas chromatography within each class.

^b^The two pear varieties were Huangjin (HJ) from *Pyrus pyrifolia* and Jimi (JM) of *Pyrus bretschneideri*. The peach variety was nectarine peach of *Prunus persica* (L.) Batsch cv. Shuguang.The two blends were based on composition of headspace VOCs from mature detached fruits (DS), intact shoots (IS).

^c^Farnesene = mixture of (*E,E*)-alpha-farnesene (49%), (*E*)-beta-farnesene (26%), (*Z*)-beta-farnesene (18%), and (Z*,E*)-alpha-farnesene (7%).

### Data analysis

Data for male and female OFM catches were analysed by one-way ANOVA, followed by the Tukey's multiple comparison test to assess the effect of different lures on catches (*P* < 0.05). Significant differences in three flight behaviours in the wind tunnel between both sexes were analysed by Mann–Whitney *U*-tests. ANOVA was also used to compared the attractiveness of mated OFM females and males to the VOC source in the wind tunnel. The means were separated by Tukey's multiple range tests (*P* < 0.05). All data were analysed with the statistical program SPSS (version 21.0).

## Supplementary information

Supplementary Table S1.
