# Supplementary material for: Peach volatile emission and attractiveness of different host plant volatiles blends to Cydia molesta in adjacent peach and pear orchards
Source: Sci Rep. 2020 Aug 12;10:13658. doi: 10.1038/s41598-020-70685-9 (PMC7423959; doi:10.1038/s41598-020-70685-9)
Supplement: Supplementary file 1 — Supplementary Table S1. [file 41598_2020_70685_MOESM1_ESM.doc]

Peach volatile emission and attractiveness of different host plant blends to *cydia molesta* in adjacent peach and pear orchards

**Peng-fei Lu^1^*and Hai-li Qiao^2^**

^1^ The Key Laboratory for Silviculture and Conservation of the Ministry of Education, School of Forestry, Beijing Forestry University, Beijing 100083, China

^2^ Institute of Medicinal Plant Development, Chinese Academy of Medical Sciences and Peking Union Medical College, Beijing, 100193, China

*****Correspondence: lpengfei224@126.com; Tel.: +86-10-6233-6755

Supplementary Table S1 Glossary of abbreviations for different plant treatments in the manuscript

|  | **Abbreviation** | **Definition** |
| --- | --- | --- |
| **Detached**  **peach**  **mimic blends^a^** | DS | Detached peach shoots |
|  | DF | Detached mature peach fruits |
|  | DFU | Detached unmatured peach fruits |
|  | DSC | Common components released by both DS and DF, using the component ratio typical for DS |
|  | DFC | Common components released by both DS and DF, using the component ratio typical for DF |
|  | DSS | VOCs selectively released from DS |
|  | DFS | VOCs selectively released from DF |
| **Intact**  **Peach**  **mimic blends^a^** | IS | Intact peach shoots |
|  | IF | Intact matured peach fruits |
|  | IFU | Intact unmatured peach fruits |
| **Living**  **peach tissue^a^** | L-DS | Living detached peach shoots (ca. 220 g, 40 cm long) |
|  | L-DF | Living detached peach fruits (ca. 220 g, 8-10 cm diam.) |
|  | L-IS | Living intact young peach shoots tips from one-year-old potted peach plants (ca. 220 g, 40 cm long) |
| **Detached**  **Pear**  **mimic blends^b^** | JM | Detached mature pear fruit derived from JM variety |
|  | HJ | Detached mature pear fruit derived from HJ variety |

^a^Peach varieties were nectarine peach of *Prunus persica* (L.) Batsch cv. Shuguang.

^b^Two pear varieties were Huangjin (HJ) from *Pyrus pyrifolia* and Jimi (JM) of *Pyrus bretschneideri*.
